# Rare early prosthesis obstruction after mitral valve replacement: a case report and literature review

**DOI:** 10.1186/1749-8090-7-64

**Published:** 2012-07-02

**Authors:** Jun Shi, Zhi-xuan Bai, Jia Hu, Ben-gui Zhang, Ying-qiang Guo

**Affiliations:** 1 Department of Cardiovascular Surgery, West China Hospital, Sichuan University, Cheng Du, People’s Republic of China

## Abstract

As a dreadful complication after the mechanical heart valve replacement, prosthetic valve obstruction caused by pannus formation occurs increasingly with time. The authors here present a case of 42-year-old woman who was urgently admitted to hospital with acute heart failure symptoms due to the mechanical mitral valve failure only 3 months after surgery. Transthoracic and transesophageal echocardiography demonstrated that the bileaflet of the mitral prosthesis were completely immobilized with only a small transvalvular jet remained. During the reoperation, the reason of the prosthetic valve obstruction was attributed to the noncircular pannus ingrowth extending from the atrioventricular side. For a better understanding of the prosthetic valve dysfunction caused by pannus formation, the authors then compile a literature review to briefly discuss the status quo of the clinical characteristics of this uncommon complication.

## Background

The evolving design and biomaterials of the mechanical heart valves have greatly advanced their *in vivo* hemodynamic features and durability over decades [1]. However, the anticipated performances of the mechanical prostheses are still compromised by the occurrence of various complications, among which pannus-induced prosthetic valve dysfunction (PVD) is relatively uncommon but sometimes is the most serious one [2,3]. In particular, patients with prosthetic valve obstruction (PVO) due to pannus ingrowth may rapidly develop hemodynamic deterioration and crash into a life-threatening condition. According to recent studies [2,4-7], the prevalence of pannus formation in aortic or mitral position is controversial. However, pannus is undoubtedly of later clinical onset than the thrombosis, which is mostly responsible for the early PVD. Moreover, the duration from time of prostheses implantation to pannus-induced PVD is widely variable and has been reported to be at least 6 months to 12 months, during which an ingrowth of periannular tissue would gradually immobilize the moving element of the prostheses [6-8]. Here we present a rare case of PVO in the mitral position caused by early pannus formation only 3 months after surgery.

## Case presentation

A 42-year-old woman was admitted to our intensive cardiac care unit with symptoms of acute left heart failure. Three months prior to admission, she underwent the implantation of a mechanical bileaflet mitral valve (25 mm SJM Master; St. Paul, MN, USA) due to severe rheumatic valve stenosis. No native valve- or chordal-sparing procedures were performed during the replacement. The postoperative course went smoothly and transthoracic echocardiography (TTE) showed proper functioning of the prosthesis. The patient then followed adequate anticoagulation treatment with wafarin and the international normalized ratio maintained above 2.0. No episode of atrial fibrillation and other risk factors for thrombus formation were identified. One week before this admission, she had noticed a progressive physical deterioration. After the treatment at local hospital, the patient showed no signs of recovery and finally presented with severe shortness of breath and coughing up pink, foamy mucus on the arrival of our hospital.

**Figure 1:**
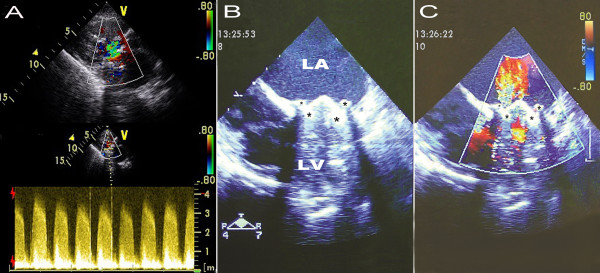
**Echocardiography of mitral prosthesis: A) Transthoracic echo reveals immobilization of the mechanical bileaflet with accelerated velocity of transvalvular blood flow; B) and C) Intraoperative transesophageal echo demonstrated prosthetic valve obstruction due to pannus formation (asterisks) on atrioventricular side.** LA indicates left atrium; LV indicates left ventricle.

On admission, the patient was conscious but presented with marked respiratory distress. Initial vital signs showed blood pressure 80/40 mmHg, sinus rhythm, a regular pulse of 140 beats/min and a respiratory rate of 40 breaths/min. Auscultation of the chest revealed diffuse rhonchi and expiratory wheezes without any distinct heart murmurs. No signs of jugular venous distention and extremities edema were observed. The patient was intubated due to the deteriorated respiratory status. After intubation, bedside TTE demonstrated that the prosthetic bileaflet was immobilized and only a small transvalvular jet was observed. However, no paravalvular leakage, and vegetations or periannular abscesses that indicating endocarditis were identified (Figure 1A).

**Figure 2:**
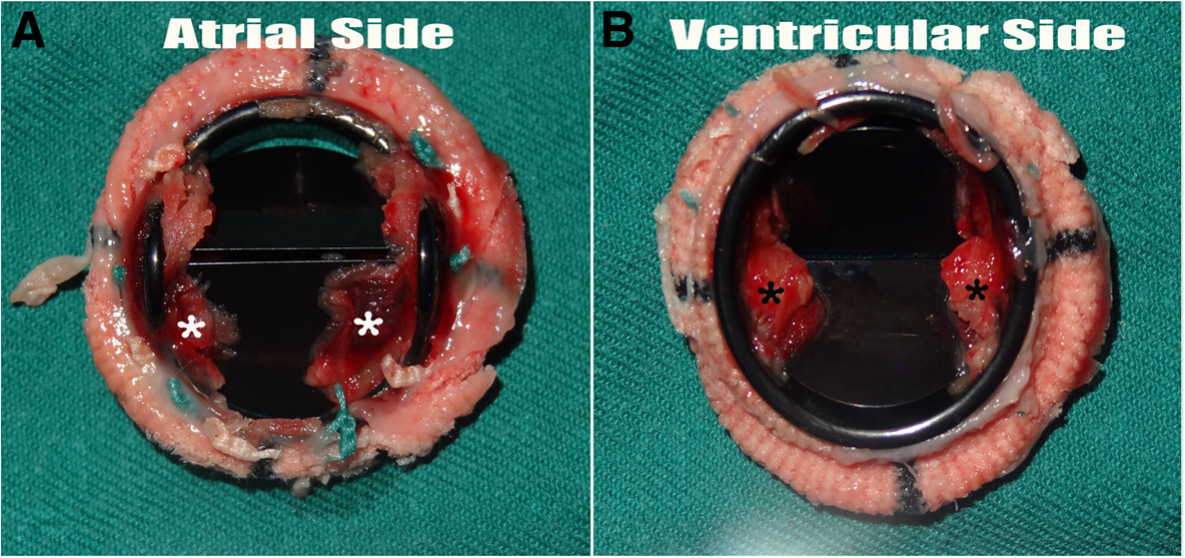
A macroscopic view of explanted St. Jude prosthetic valve with pannus ingrowth (asterisks): A) Atrial aspect; B) Ventricular aspect.

In view of the patient’s unstable hemodynamic conditions and echocardiographic findings, an emergent surgical intervention was performed. Intraoperative transesophageal echocardiography (TEE) demonstrated the immobilization of the prosthetic leaflets, which might be attributed to the acoustic shadowing around the valve ring **(**Figure 1B and Figure 1C**).** During surgical inspection, a noncircular fibrotic tissue ingrowth from the atrioventricular side was detected. The invaded pannus strictly adhered to the valve pivots and arrested both prosthetic leaflets (Figure 2). The failed prosthesis was explanted and a new 27 mm SJM Master mechanical valve was implanted. The postoperative course was regular and in-hospital TTE showed normal functioning of the newly implanted mitral prosthesis. The patient had an uneventful recovery and was discharged home at two weeks after surgery.

**Figure 3:**
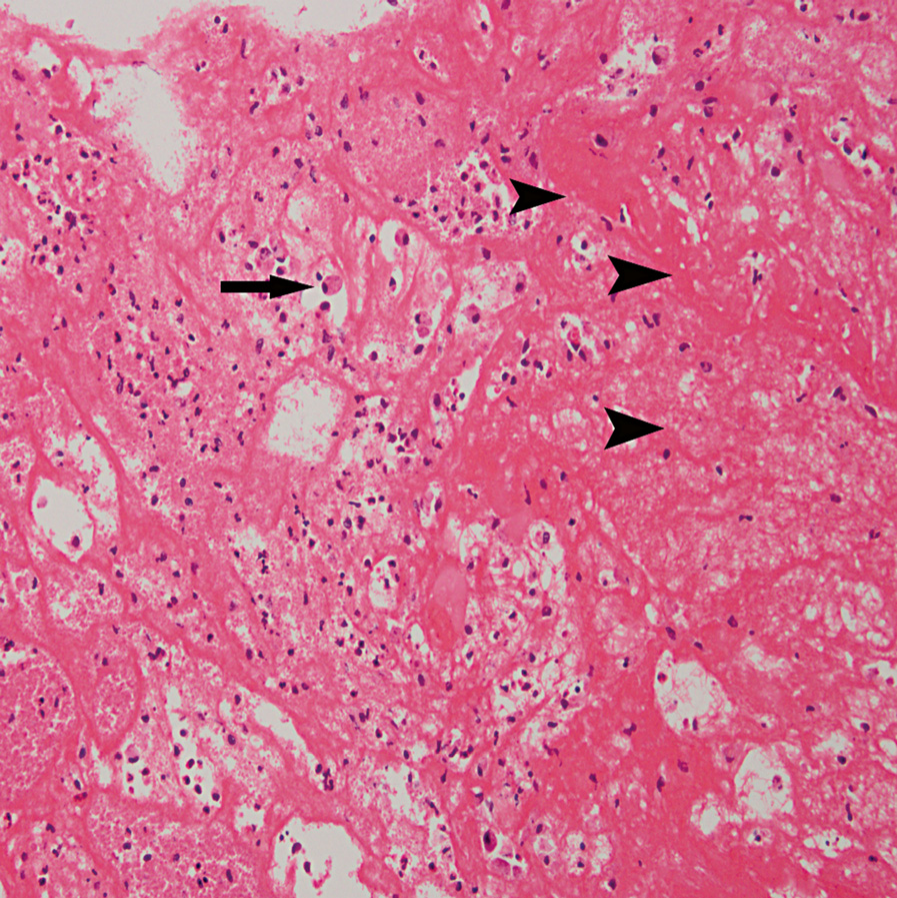
**Histologic section shows that the specimen largely consist of infiltrated leukocytes (arrow) and pleomorphic spindle cells such as myofibroblasts (arrowheads), as well as interspersed capillary vessels.** Magnification × 200.

On microscopic examination (Figure 3), the resected pannus tissue was found to be mainly constituted with infiltrated leukocytes (neutrophils, macrophages, lymphocytes and plasma cells), pleomorphic spindle cells such as myofibroblasts, and interspersed capillary vessels.

## Discussion

As an exuberant healing process in response to the implanted prosthesis, pannus formation occurs increasingly with time. Compared with valvular thrombosis, pannus is less frequently found to be an etiologic factor that contributes to the early PVD [2-8]. According to multiple clinical observations (Table 1), the duration from the time of prostheses implantation to pannus-induced PVD varies widely and has been reported to be 1.8 years to 23 years after surgery [4-7,9,10], and a shorter time interval (6 months) has been described in patient with the prosthetic aortic valve [8]. However, in this case, the time interval from the initial replacement to PVO caused by pannus ingrowth was extraordinarily short. To our best knowledge, there were no reports of pannus-induced PVO within one year after mitral valve replacement [11].

**Table 1:** Studies of mechanical prosthetic valves obstruction due to pannus formation

**Authors, years, [Ref.]**	**Type of studies**	**Position of Valves**	**Incidence & Time Interval**	**Risk Factors**
Vitale et al. , 1997, [4]	Case series (n = 1878)	Mitral valves	3.5%, Mean ≥ 4 years	Female, Tilting-disc valves, Bileaflet valves
Barbetseas et al. , 1998, [6]	Case series (n = 23)	Mitral &Aortic valves	More common in aortic position, 178 ± 52 months	Not indicated
Rizzoli et al. , 1999, [7]	Case series (n = 2680)	Mitral &Aortic &Tricuspid valves	0.24%/patient/year, Median = 13 years	Tilting-disc valves, Caged-disk/ball valves
Teshima et al. , 2003, [5]	Case series (n = 615)	Aortic valves	1.95%, Mean = 83 ± 52 months	Inadequate anticoagulation, SJM valves
Sakamoto et al. , 2006, [9]	Case series (n = 390)	Aortic valves	1.8%, Mean = 10 ± 7.9 years	Small prostheses size Turbulent Flow
Kondruweit et al. 2008, [8]	A case report	Aortic valve	6 months	Small prosthesis size, Rheumatic fever
Mullenix et al. , 2008, [3]	A case report	Aortic valve	15 years	Female, A tilting-disc valve
Hurwitz et al. , 2009, [2]	A case report	Aortic valve	8 years	Female, Endocarditis
Khan et al. , 2009, [12]	A case report	Mitral valve	7 years	Female, Subvalvular chordae preservation
Matsuyama et al. 2011, [11]	A case report	Mitral valve	27 months	Inadequate anticoagulation, female
Park et al. , 2011, [13]	A case report	Mitral valve	9 years	Female, Subvalvular chordae preservation

The cause of the pannus-related PVD and PVO is generally considered as a chronic foreign body reaction to the prosthetic biomaterials. It is likely that an individual patient’s inherent propensity with coexisting risk factors triggers and accelerates the progression of pannus formation. These associated factors can be summarized as follows: 1) Clinical characteristics of the patients: female, concomitant endocarditis and pregnancy, the history of rheumatic fever and valvular thrombosis, atrial fibrillation, low-output conditions, small valve annulus and inadequate anticoagulation [2,5-9] Design and biomaterials of the prostheses: tilting disc valves and caged-disk/ball valves due to low transvalvular flow [7,11,14], the protruding design of the pivot guard systems in the SJM valves [3,15] Surgical techniques: preservation of the native valve or subvalvular chordae [12,13], implantation of the prostheses with smaller size [9], the presence of periannular endothelial irregularities after implantation [2,4,7]. Taken together, as presented in our case, the history of severe rheumatic disease, small mitral annulus and implanted SJM prosthesis in this female patient may have contributed to the acute formation of the pannus.

The underlying mechanism of the pannus formation was supposed to be the significant proliferation and deposition of myofibroblasts, phagocytes and extracellular matrix, which are mediated by increased expression of transforming growth factor type beta-1 (TGF-β1) in perivalvular tissue and circulating blood [5,16]. Moreover, some studies have suggested that the persistent release of fibroblast growth factor-2 (FGF-2) from the injured periannular tissue may also contribute to this chronic healing process [17]. Therefore, echo to these important observations, a promising therapeutic strategy to prevent pannus formation in high risk patients might be achieved by suppressing the proliferative responses in periannular tissues with the drug-coated prostheses.

The obstruction of prosthetic valves is largely caused by pannus, thrombus or both, so it is of great therapeutic implications to make differential diagnosis on them. Because reopen heart surgery, either repeated valve replacement or resection of the ingrowth tissue [13], is the only option for managing the pannus-induced PVO, while thrombolytic therapy can serve as an alternative treatment in selected patients with thrombosed prostheses [18,19]. As reported in several echocardiographic studies, pannus in situ presents with a higher ultrasound-intensity ratio (>0.7) [6] and is usually found to be a fixed lesion that attaches to the prosthetic ring [2]. Conversely, the thrombotic mass demonstrated in echo is mobile and is generally attaching to the valve occluder [1,2,20]. Although thrombolysis may be preferred for its less invasiveness in certain group of patients, surgical intervention, as for this case, should be unhesitatingly performed once the patients show any signs of hemodynamic instability.

## Conclusions

With adequate anticoagulation and no preservation of subvalvular tissue in the first-time mitral valve replacement, the pannus-induced obstruction of an SJM bileaflet mechanical valve within such a short time interval was very uncommon. This rare case has added to a growing body of literatures describing the various clinical features of the pannus-related PVO. Further explorations on etiologic-specified prevention, and efficient treatment for this complication are required.

## Consent

Written informed consent was obtained from the patient for publication of this case presentation and accompanying images. A copy of the written consent is available for review by the Editor-in-chief of this journal.

## Competing interests

The authors declare that they have no competing interests.

## Authors’ contributions

All authors contributed equally to the manuscript and all authors read and approved the final manuscript.
